# The effect of educational gymnastics on postural control of young children

**DOI:** 10.3389/fpsyg.2022.936680

**Published:** 2022-08-10

**Authors:** Neil Anderson, Chris Button, Peter Lamb

**Affiliations:** School of Physical Education, Sport and Exercise Sciences, University of Otago, Dunedin, New Zealand

**Keywords:** motor development, gymnastics, unipedal balance, sway regularity, children, fundamental movement skills

## Abstract

Fundamental movement skill (FMS) proficiency does not develop solely due to maturation, but also *via* diverse perceptual-motor experiences across childhood. Practicing gymnastics has been shown to improve postural control. The purpose of the present study was to examine potential changes to postural control of children following a course of educational gymnastics. Two groups of children both completed 20 × 45-min physical education (PE) lessons; one group (*n* = 43, age = 6.4 ± 0.7, 56% male) completed educational gymnastics lessons in school delivered by a professional coach, the other group completed their typical PE classes (*n* = 18, age = 6.5 ± 0.3, 33% male). Unipedal balancing performance was assessed by calculating the percentage of successful trials made. Postural sway dynamics were explored by calculating center-of-pressure sample entropy, 95% ellipse sway area and sway velocity. Measurements were taken before the lessons began and immediately after the lessons were completed. The gymnastics group performed better than the typical PE group at unipedal balancing. Females outperformed males in both groups. Males made different changes to postural control (i.e., increased sway regularity and improved stability) compared to females across 3 months. Educational gymnastics enabled children in a critical period of development to make more rapid improvements to postural performance and control. Novel movement experiences, like those offered by educational gymnastics, may have a positive influence on postural control and importantly, physical literacy. Future work should examine how sex effects the development of postural control strategies in young children.

## Introduction

Fundamental movement skill (FMS) proficiency does not develop solely due to maturation, but also *via* diverse perceptual-motor experiences across childhood (Okely et al., 2001; Clark, 2007; Stodden et al., 2008; Haywood and Getchell, 2009). The development and performance of FMS is constrained in particular by postural control (Woollacott et al., 1987; Assaiante, 1998; Clark, 2007; Cluff et al., 2010). However, physical maturation also has a role in the development of motor skills including posture, such that females typically develop enhanced postural skills earlier than males (Neves et al., 2021). Indeed, between the ages of approximately 6-and 8-years old children enter a critical period within which they need to recalibrate postural control to accommodate for rapidly changing body size and maturation of the perceptual-motor system (Riach and Starkes, 1994; Assaiante and Amblard, 1995; Kirshenbaum et al., 2001; Austad and van der Meer, 2007). Within this critical developmental period, postural performance can be compromised (Rival et al., 2005).

### Development of static postural control

To help understand how humans control their posture, body sway measures such as center-of-pressure (COP) deviations can be obtained from force plate measurements (Kirshenbaum et al., 2001). Two common linear variables reported in the literature include: sway path velocity and sway area (Ladislao and Fioretti, 2007; Zumbrunn et al., 2011); such variables can be described as “linear” as they are based on linear assumptions of the dataset such as central tendency (Anderson and Button, 2017). The size of the COP sway area is assumed to reflect an individual’s ability to integrate perceptual-motor information to control a postural task effectively with smaller sizes corresponding to better postural stability and control (Fitzgerald et al., 1994; Garcia et al., 2011). Changes to sway area fluctuations can result from increasing age, training, or changes in environmental conditions (Riach and Hayes, 1987; Figura et al., 1991). In typically developing people, the sway area of the COP tends to decrease from birth until adulthood, then increase with age (Newell et al., 1997; Fujita et al., 2005; Hsu et al., 2009).

COP velocity may reveal more about the control processes used to regulate posture (Kirshenbaum et al., 2001; Davids et al., 2008); fast movement of the COP employs open loop control strategies and is less precise (Kirshenbaum et al., 2001). Whereas slower COP movement uses perceptual-motor information for more precise guidance (Kirshenbaum et al., 2001). Typically, younger children use quick, ballistic movements of center of pressure to control posture and at around age eight or nine they begin to make slower movements of center of pressure indicating a transition to an integrated open-closed loop mode of control that allows for more effective postural control (Kirshenbaum et al., 2001).

Nonlinear variables provide researchers with novel insight into how performers adapt posture as a function of increasing age, expertise or different postural challenges (for more details see: Anderson and Button, 2017). Compared to linear sway variables, nonlinear analysis techniques are arguably more sensitive to postural control differences revealed under various conditions (Newell, 1998; Harbourne and Stergiou, 2003; Cavanaugh et al., 2006; Stins et al., 2009). Nonlinear variables such as sample entropy are often used to capture the stochastic dynamics of human movement (Newell, 1998). Sample entropy (SE) quantifies the complexity in time series data, such as COP, and it helps depict the regularity of human behavior during stance (Newell, 1998; Richman and Moorman, 2000; Cavanaugh et al., 2005). Low SE values are indicative of a more regular pattern (e.g., similar to a sine wave) in the time series data and higher SE values indicate stochastic, or random fluctuations in the time series (akin to white noise; Cavanaugh et al., 2005). Indeed, Newell (1998) suggested that increased complexity is a reflection of additional degrees of freedom contributing to the control of posture. Whereas “regular” time series data can be found in situations when posture is challenged such as during sensory deprivation or alterations to the base of support (i.e., standing compared to sitting). Conversely, “irregular” sway has been suggested to indicate high postural automaticity, expertise in postural tasks and increased postural stability (i.e., sitting compared to standing; Pincus and Goldberger, 1994; Roerdink et al., 2011).

It is unclear how COP sway regularity during quiet stance changes with age from the extant literature (Newell, 1998; Schärli et al., 2012). Newell (1998) reported an inverted U-shaped trend in the approximate entropy (ApEn) of COP such that from childhood (approximately 3-year-old) into adulthood, regularity decreased and then increased into old age. However, using sample entropy (SampEn), Schärli et al. (2012) found different results compared to Newell (1998). Schärli et al. (2012) reported an increase in COP sway regularity from childhood (*n* = 15, 5-, 8- and 11-year-old) to adulthood (*n* = 15) in quiet standing with a fixed gaze. It is challenging to explain the discrepancy between the results of Newell (1998) and Schärli et al. (2012) which may result from a combination of factors, i.e., the different type of entropy calculated (i.e., ApEn or SampEn), the parameters used when calculating the respective entropy statistic (e.g., tolerance for matches or the length of pattern to be matched), the length of trials in each study (i.e., 15 and 30 s), the nature of the task demands (e.g., participants in the Schärli et al. (2012) study fixed their gaze on a target) or the lack of an elderly group in Schärli et al’s research. Hence, more research is needed to help clarify how sway regularity changes with increasing age.

It is also of interest to consider how postural control is maintained under more challenging constraints than quiet standing (e.g., unipedal balancing). While some have reported that with increasing age, unipedal balance performance has been shown to improve (Morris et al., 1982; Condon and Cremin, 2014), Clark and Watkins (1984) found no difference between two groups of children (mean ages 6.7 and 7.7 years). When investigating linear COP variables, sway area and velocity have been reported to reduce with age when children are asked to stand on one foot (Figura et al., 1991; Mickle et al., 2011; Zumbrunn et al., 2011). Interestingly, investigations into the influence of sex on unipedal balance performance have been somewhat inconclusive: some literature reports that from approximately the age of five females performed better in balance tests compared to males (Morris et al., 1982; Raudsepp and Pääsuke, 1995; Venetsanou and Kambas, 2011), whereas others failed to find differences in balance performance between sexes (Junaid and Fellowes, 2006) or did not mention the effect of sex in their analysis (Zumbrunn et al., 2011). When considering the kinematics of unipedal balance, Figura et al. (1991) reported no effect of sex on sway velocity or range, whereas Lee and Wei-Hsiu (2007) and Mickle et al. (2011) reported that females swayed over a smaller area compared to males.

Only one study to our knowledge has compared sway regularity in unipedal balancing by young children. Guimarães-Ribeiro et al. (2014) showed young females (mean age of 9.6-year-old) swayed significantly less regularly in the anterior posterior (A/P) direction compared to middle aged women (46.6-year-old) but not in the mediolateral (M/L) direction. Guimarães-Ribeiro et al. (2014) concluded that the young females had a more automatic and more efficient postural sway. To our knowledge, no other research has investigated the effect of increasing age or sex on sway regularity in unipedal balancing.

Along with age, specialist training also influences postural sway regularity in quiet standing (Lamoth et al., 2009; Stins et al., 2009; Isableu et al., 2017). Lamoth et al. (2009) compared gymnastically trained university students (mean age 21.3 years) to other university students (including physical education students; mean age 20.6 years) and found that increased gymnastic skill resulted in less regular postural sway. Similar results were found by Stins et al. (2009), albeit in a younger cohort (mean age 12.4 years), where dancers had less regular sway compared to non-dancers. Under more challenging conditions (i.e., quiet stance with eyes closed), Isableu et al. (2017) found that gymnasts had less regular sway compared to non-gymnasts. Indeed, practicing gymnastics (either recreationally or competitively) has been shown to improve FMS and postural skills (Garcia et al., 2011; Karachle et al., 2017; Rudd et al., 2017a,b). Garcia et al. (2011) found in their study that young children (aged 5–7 years) who practiced gymnastics had developed better postural control compared to non-gymnasts. In the study by Karachle et al. (2017) children (*n* = 34, mean age 4.7 ± 1.2 years) completed two recreational gymnastics lessons per week for 6 months resulting in higher motor proficiency (measured using the Bruininks-Oseretsky Test of Motor Proficiency, 2nd edition (BOTM-2)) compared to a control group. Rudd et al. (2017a,b) employed school-based gymnastics interventions of up to 16 weeks and showed that years 2 and 4 of primary school children made larger improvements to FMS (measured using the Test of Gross Motor Development, 2nd Edition) compared to control groups. Importantly, while improvements to FMS were shown, none of these gymnastics intervention studies directly investigated the effects of the intervention on postural control.

### The present study

It appears that exposure to educational gymnastics may assist with the development of postural control of young children. The analysis of COP, collected during static unipedal stance, can discriminate postural control strategies between training groups, and other influential factors including sex and age. The aims of the present research were to: (1) determine how gymnastics training affected the longitudinal development of static postural performance and control in young children (between approximately 5- and 8-year-old); (2) investigate how COP sway kinematics change with changes in unipedal balance performance and (3) identify differences between sexes in postural control following gymnastics training.

It was predicted that children receiving bi-weekly gymnastics training would perform better and improve more at balance performance compared to the Typical Physical Education “TPE” group. Additionally, it was predicted that children that practiced gymnastics would make larger changes to COP variables compared to the TPE group. Finally, it was predicted that females’ balance performance would be better than males and that males and females would make different changes to sway kinematics.

## Materials and methods

### Participants

Two primary schools agreed to allow a specialist, trained educational gymnastics coach lead year-two classes through bi-weekly 45-min lessons for one school term (10-weeks) in place of the regular physical education programme. Two other schools participated as controls in which they continued to provide their regular physical education curriculum. Due to the logistics of delivering the gymnastics programme, participants could not be randomly assigned to groups. Consequently, we were unable to recruit as many participants to the control group (*N* = 18) as parents generally preferred for their children to take part in the intervention programme (*N* = 43). The control schools were matched to the intervention schools for socioeconomic status and locations (i.e., suburban schools in a small city). From this point on these groups will be referred to as the “GYM” and “TPE” groups, respectively. Any child with sensory-motor or musculoskeletal disorders was excluded from the study. Children were also excluded if they had previously practiced any form of gymnastics for more than 3 months (one student). Three children allocated to the GYM dropped out due to their families moving from the area during the study. All children in the TPE group completed each phase of the study. Children and their parents/caregivers completed consent forms prior to engaging in the educational gymnastics class or data collection process. Table 1 presents information about the children in each group.

**Table 1:** Gymnastics and TPE group participants’ age and sex at each phase of data collection.

	Gymnastics		TPE	
Phase	1	2	1	2
Participant numbers (males/females)	24/19	24/19	6/12	6/12
Age (years ± SD)	6.4 ± 0.7	6.7 ± 0.7	6.5 ± 0.3	6.8 ± 0.3

### Equipment

Center-of-pressure (COP) displacement was measured using an AMTI (Watertown, MA) force plate (model OR6-5-1) and strain gage amplifier (model SGA6-4). Gain for the force plate was set at 1,000. All GRF data were captured using Vicon Nexus 1.8.5 software (Vicon, Oxford, United Kingdom) at 1000 Hz. A high-speed video camera (Basler, model-piA640-210gc, Ahrensburg, Germany) operating at 200 Hz was used to film children performing the tasks. Video footage synchronized with the force plate data was used to determine the start, finish and number of foot touch downs in the balancing task. A 50-inch television (Sony Bravia) displayed images of static cartoon characters for children to look at while they balanced. The television screen was positioned 4 m from the children at eye line.

### Procedure

A 3-month longitudinal, mixed methods experimental design was employed. At the first visit to the laboratory, parents and children were asked to complete consent forms. Before any kinematic or performance measurements were made, children had a familiarization session in which they practiced the activities of the testing protocol.

Data collection consisted of two tests (phases) containing the same assessment procedures. Phase one was used to obtain baseline data and was completed in the school holidays before the start of term one of the school year. Following the phase one data collection, each group completed bi-weekly physical education classes for 10 weeks (the length of the school term). The GYM group participated in specially designed educational gymnastics lessons; these lessons were taught by a qualified gymnastics coach and incorporated basic artistic and rhythmic gymnastics skills. The TPE completed their normal primary school physical education under the instruction of their usual teacher who was a trained physical education teacher. After the first school term had finished, participants were invited back to the laboratory to complete phase two.

Static unipedal balance ability was assessed using the static balance test from the Movement Assessment Battery for Children – 2 (MABC-2). In the present study, participants were asked to stand quietly on a force platform on one foot for a period of 20 s. Participants alternated between balancing on the dominant and non-dominant foot and they were asked to look at a target (cartoon monster displayed on the TV screen) to minimize visual wandering. After 20 s a red “stop” sign was displayed on the television and children could cease balancing on one foot. Participants were asked to try to avoid touching the non-supporting leg on the ground or against the supporting leg. The number of “errors,” i.e., touches by the non-supporting leg on the floor or against the support leg for maintenance of balance, were counted by the experimenter. Participants were told that they could move their arms how they wished to maintain balance. This test was repeated up to five times per foot. Ground reaction force data collection was commenced at least 5 s before the children began balancing on one foot. The high-speed camera was located directly behind the participants to enable visual detection of errors during each trial.

### Data analysis

Before further data processing, trials that did not satisfy the task’s constraints were identified (e.g., shuffling on the support leg, briefly touching the non-support leg on the floor or hopping to maintain balance). Further, static postural control trials that were not successful (i.e., when an error was made) were excluded from the kinematic analysis, but were included in the performance outcome analysis, specifically to allow for the calculation of the percentage of successful trials. Balance performance (i.e., % success) was calculated by dividing the number of successful trials by the number of trials attempted and expressing the result as a percentage. A trial was determined to be successful if no errors (e.g., touchdowns, shuffles or hops on the support leg, or the non-support leg touching the support leg) were made during the 20 s of unipedal balancing.

COP data were processed and analyzed using MATLAB R2011a (version-7.12.0.635, MathWorks, Natick, MA) and custom designed scripts. Data were cropped so that the end of the trial was defined as the frame when the “stop” sign appeared on the TV (identified by watching the recordings) and the first frame occurred 20 s earlier. A zero-lag, 4th order low-pass Butterworth filter with a 10 Hz cut off frequency was applied to the data and the length of trials were normalized to 2000 points.

Sample entropy, sway area and sway velocity were calculated from the processed (M/L and A/P) COP data using the MATLAB *SampEn* (Lee, 2012), *COPellipse* (Duarte and Zatsiorsky, 2002) functions and custom written script, respectively. The following parameters were used in the sample entropy calculation: sequence length for matching, *m* = 2 and tolerance for matching, *r* = 0.2* SD (Caballero et al., 2015). COP sway area was calculated as the size of the ellipse that covered 95% of points in the COP sway.

Sway velocity was calculated by dividing the total distance traveled in each trial by the COP by the time taken to complete each trial.

#### Statistical analysis

All statistical analyses were performed using R (version 4.1.1; R Core Team, 2021). To account for the unequal group sizes, linear mixed-effects models were fitted for each of the performance and kinematic variables using the maximum likelihood method (Bates et al., 2015), using the “*nlme*” package (Pinheiro et al., 2022). The same random and fixed effects were used in fitting each model to allow for the interactions of interest to be explored for each dependent variable. The participants were included as random effects and fixed effects were: (1) phase of the study, (2) group (GYM or TPE), (3) sex, and (4) the foot used (for performance of the task). Tests for normality and homogeneity of variance were conducted on the final models of the dependent variables using a Shapiro-Wilks test and Levene’s test, respectively, with significance levels set at alpha = 0.05. Dependent variables that failed normality and variance tests were log transformed before further analysis.

ANOVAs were performed for each dependent variable to examine the effect of fixed factors and interactions between fixed factors on the dependent variables. Factors included in the ANOVA were group (GYM or TPE), phase (repeated data collection) and sex (male or female) and foot used for balancing. Estimated marginal means were calculated for dependent variables to allow for examination of the main factors influencing performance and postural kinematics. When main or interaction effects were found *post hoc* pairwise comparisons were performed (alpha = 0.05) on estimated marginal means, using the “*contrast”* and “*test*” functions, to compare between groups, phases and sexes and foot used. Bonferroni corrections were made to account for multiple comparisons. *Post hoc* tests are reported as t-ratios. Distributions within group and sex for each dependent variables are shown with boxplots. To quantify the size of the differences between phases, groups or sexes, Cohen’s *d* was calculated with reference values for small, medium and large sized differences set to 0.2, 0.5 and 0.8, respectively, as indicated by Cohen (1988).

## Results

Table 2 provides a summary of significant main effects and interactions for the study. Preliminary ANOVAs revealed no interaction effect between foot used for balancing and any of the other factors for any of the dependent variables and as such data were averaged across feet for further analysis.

**Table 2:** Summary of significant ANOVA results for balance performance and kinematic variables.

	df	*F*-score	Value of *p*
*% Success*			
Phase	1, 169	8.07	0.005
Sex	1, 57	5.34	0.024
*A/P SampEn*			
Sex	1, 56	6.48	0.014
Phase:Sex	1, 555	8.45	0.004
*M/L SampEn*			
Sex	1, 56	4.43	0.040
Phase:Sex	1, 555	4.79	0.029
*95% Ellipse Area*			
Phase	1, 555	6.86	0.009
Phase:Group	1, 555	7.00	0.008
Phase:Group:Sex	1, 555	4.73	0.003
*A/P Velocity*			
Phase	1, 555	7.26	0.007
Sex	1, 56	6.32	0.015
Phase:Group	1, 555	4.72	0.030
Phase:Group:Sex	1, 555	5.62	0.018
*M/L Velocity*			
Phase	1, 555	6.09	0.014
Sex	1, 56	6.67	0.013
Phase:Group:Sex	1, 555	5.52	0.019

### Unipedal balance performance

**Figure 1:**
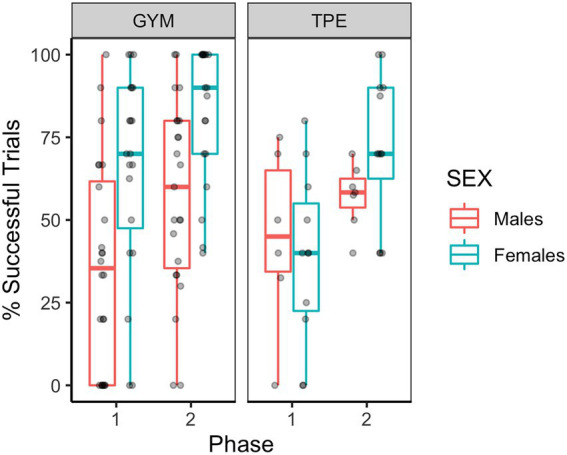
Successful trials (%) for males and females in the GYM and TPE groups.

Figure 1 shows the balance performance at phases one and two. Main effects of phase and sex were found, that is, participants improved balance performance in phase 2 (see Figure 1 and Table 2) and females were more successful compared to males (see Figure 1 and Table 2).

### Unipedal balance kinematics

#### Anterior posterior sample entropy

**Figure 2:**
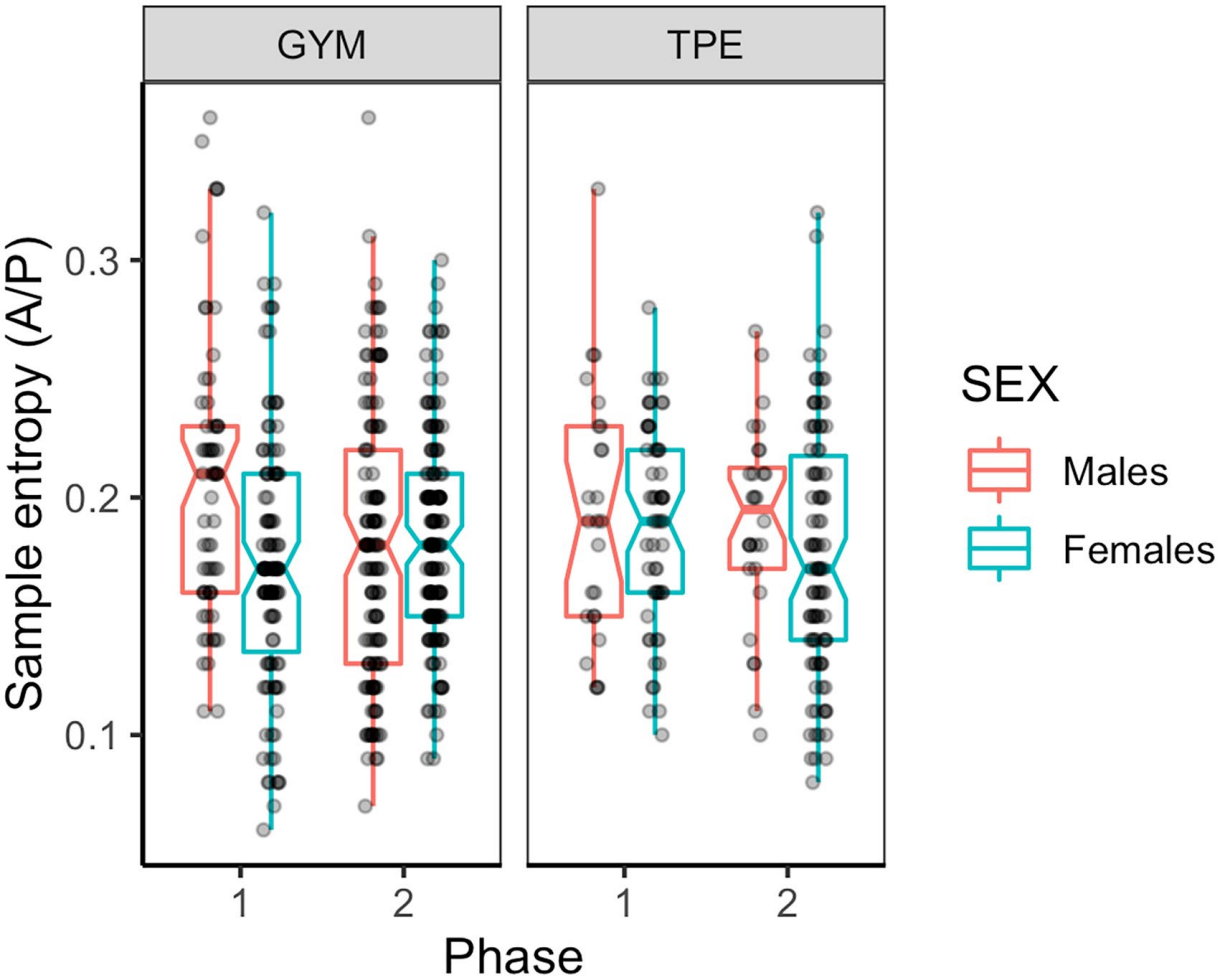
Anterior – posterior sample entropy for males and females in the GYM and TPE groups.

Figure 2 shows the A/P sample entropy during unipedal balancing by the males and females in the GYM and TPE groups across the duration of the study. A main effect of sex was found (Table 2).

An interaction between phase and sex was found (see Table 2 and Figure 2) and *post hoc* tests showed that males swayed more irregularly compared to females at phase one [*t*(555) = 2.37, *p* = 0.011, *d* = 0.19]. From phase one to two, males significantly increased sway regularity [*t*(555) = 3.67, *p* < 0.001, *d* = 0.46]. The increase to sway regularity made by males between phases one and two was larger than that made by the females [*t*(555) = 3.18, *p* = 0.015, *d* = 0.26].

#### Mediolateral sample entropy

**Figure 3:**
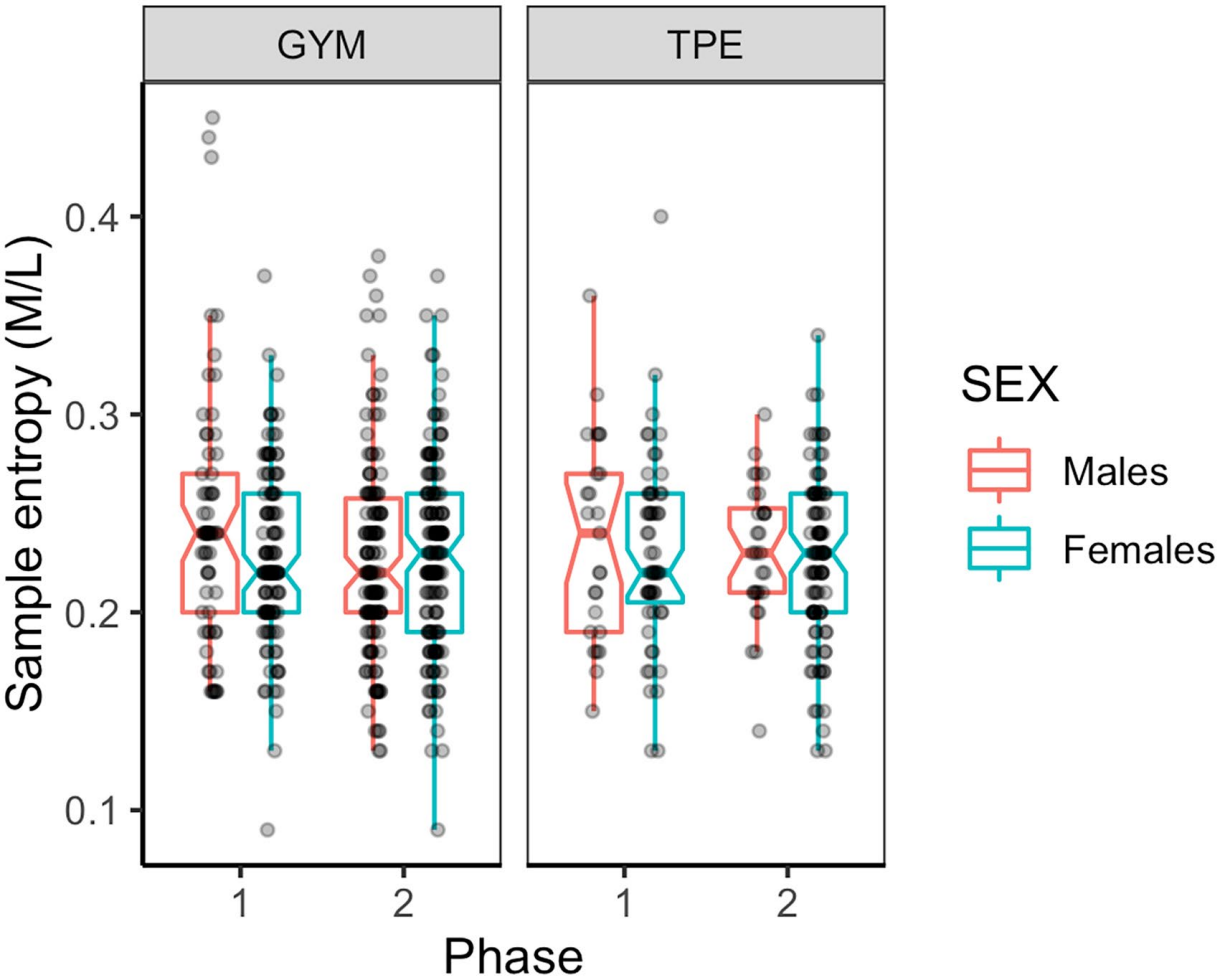
Mediolateral sample entropy for males and females in the GYM and TPE groups.

Figure 3 shows the M/L sample entropy during unipedal balancing by the males and females in the GYM and TPE groups across the duration of the study. A main effect of sex was found indicating that females swayed more regularly in the M/L direction (see Table 2). An interaction effect was found between phase and sex such that males swayed more regularly at phase two [*t*(555) = 2.47, *p* = 0.007, *d* = 0.39].

#### COP sway area

**Figure 4:**
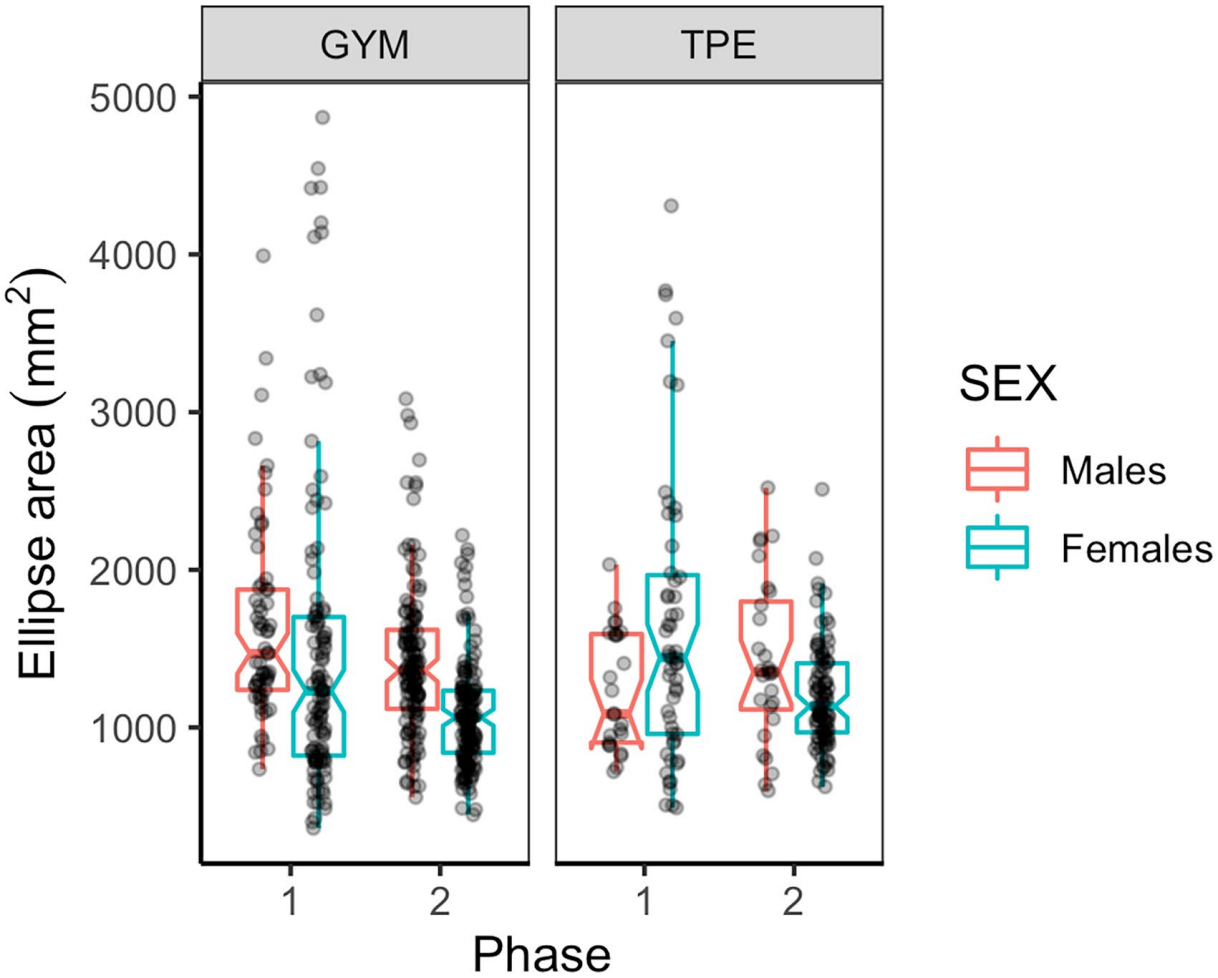
COP sway area for males and females in the GYM and TPE groups.

A main effect of phase was detected for COP sway area (Table 2). Figure 4 shows COP sway area for males and females in the GYM and TPE groups. Sway area was reduced from phase one to two. Interaction effects were revealed between phase and group and between phase, group and sex (Table 2).

*Post hoc* tests investigating the interaction between phase and group revealed that while there were no significant differences in sway area between groups at either stage of the study, the GYM and TPE groups significantly reduced sway area from phase one to two [GYM: *t*(555) = 5.20, *p* < 0.001, *d* = 0.50; TPE: *t*(555) = 1.70, *p* = 0.045, *d* = 0.25] and this reduction was larger than the TPE group [*t*(555) = 2.12, *p* = 0.034, *d* = 0.18].

When investigating the interaction between phase, group and sex, we found that at phase one, GYM males swayed over a larger area compared to TPE males [*t*(56) = 2.43, *p* = 0.009, *d =* 0.51]. Interestingly, TPE females swayed over a larger area compared to their male group mates at phase one [*t*(56) = 1.90, *p* = 0.031, *d* = 0.46]. At phase two GYM males swayed over a larger area compared to their female group mates [*t*(56) = 3.56, *p* < 0.001, *d* = 0.57]. Males in the GYM group reduced sway area between phases one and two [*t*(555) = 2.80, *p* = 0.003, *d* = 0.43], and this reduction was larger than that made by the TPE group males [*t*(555) = 3.15, *p* = 0.002, *d* = 0.49]. Females in both groups reduced sway from phase one to two [GYM: *t*(555) = 4.70, *p* < 0.001, *d* = 0.60. TPE: *t*(555) = 3.57, *p* < 0.001, *d* = 0.63].

#### Anterior posterior sway velocity

**Figure 5:**
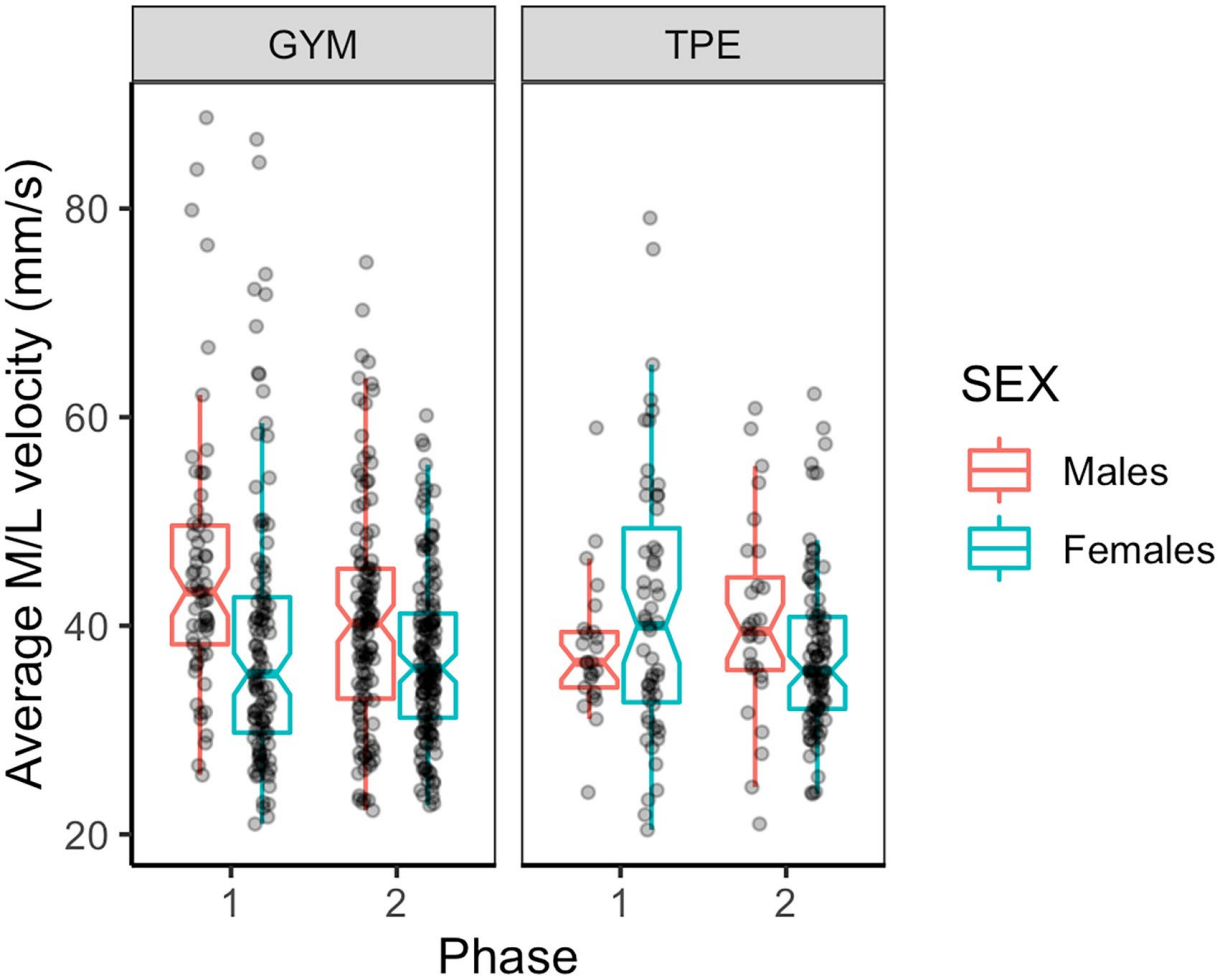
Anterior – posterior sway velocity for males and females in the GYM and TPE groups.

Figure 5 shows A/P sway velocity for males and females in each group. Main effects of phase and sex were revealed by ANOVAs where sway velocity slowed from phase one to phase two and females swayed slower than males (Table 2). Interactions between phase and group and between phase, group and sex were revealed.

When exploring the interaction between phase and group, *post hoc* tests revealed that the GYM group reduced A/P sway velocity [*t*(555) = 4.55, *p* < 0.001, *d* = 0.60] and this reduction to velocity was a significantly larger reduction than that made by the TPE group [*t*(555) = 2.23, *p* = 0.026, *d* = 0.40].

In the A/P direction an interaction between phase, group and sex was found. GYM group males swayed faster in the A/P direction than the males in the TPE group at phase one [*t*(56) = 1.95, *p* = 0.028, *d* = 0.41]. While both GYM males and females reduced A/P sway velocity between phases one and two [Males, *t*(555) = 3.81, *p* < 0.001, *d* = 0.60. Females, *t*(555) = 2.48, *p* = 0.007, *d* = 0.37], the GYM males swayed faster at phase one and two [P1: *t*(56) = 3.11, *p* = 0.002, *d* = 0.50. P2: *t*(56) = 2.49, *p* = 0.008, *d* = 0.31]. TPE group females made reductions to A/P sway velocity between phases one and two [*t*(555) = 2.74, *p* = 0.003, *d* = 0.41]. The GYM males reduced sway more than the TPE males [*t*(555) = 3.40, *p* < 0.007, *d* = 0.59].

#### Mediolateral sway velocity

**Figure 6:**
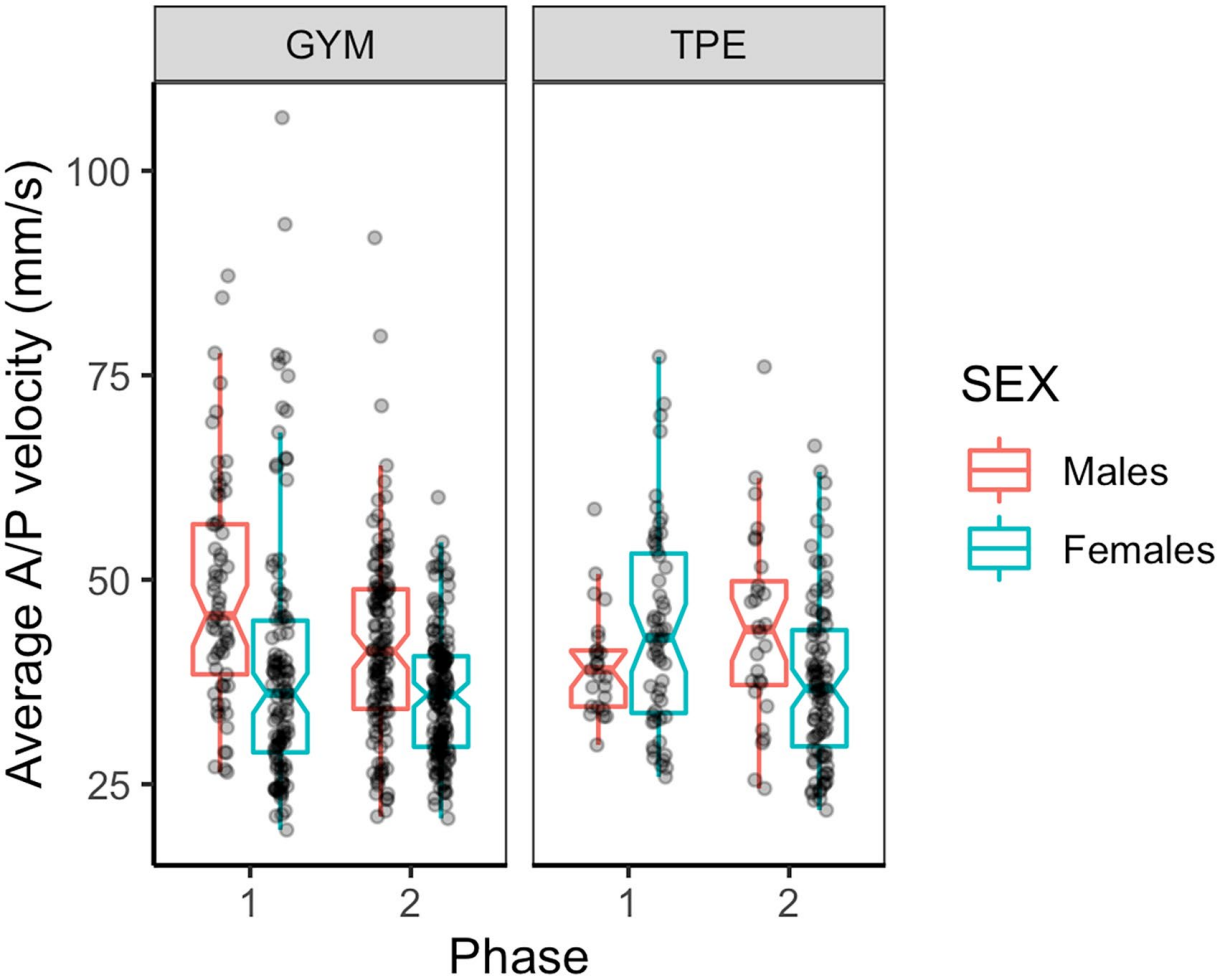
Mediolateral sway velocity for males and females in the GYM and TPE groups.

Figure 6 shows M/L sway velocity for males and females in each group. Main effects of phase and sex were revealed by ANOVAs where sway slowed across time and females swayed slower than males (Table 2). An interaction effect between phase, group and sex was found (Table 2).

*Post hoc* tests revealed that at phase one the GYM males swayed significantly faster than the TPE males [*t*(56) = 2.05, *p* = 0.022, *d* = 0.49] and their female group members [*t*(56) = 2.59, *p* = 0.006, *d* = 0.42]. GYM males also swayed faster compared to their female group mates at phase two [*t*(56) = 1.77, *p* = 0.041, *d* = 0.22]. In the GYM group, males and females reduced M/L sway velocity between phases one and two [Males: *t*(555) = 2.99, *p* = 0.002, *d* = 0.47. Females: *t*(555) = 1.73, *p* = 0.042, *d* = 0.22]. Females in the TPE group reduced sway velocity between phases one and two [*t*(555) = 2.64, *p* = 0.001, *d* = 0.46].

## Discussion

Gymnastics has been proposed as a form of physical training that can assist the development of postural skills (Garcia et al., 2011; Karachle et al., 2017; Rudd et al., 2017a,b). It was predicted that (1) gymnastically trained children would exhibit better balance performance and (2) make larger improvements to performance compared to the TPE group. We predicted that (3) the gymnastically trained children would make larger changes to sway kinematics compared to the TPE group. Finally, we expected (4) females to outperform males and (5) make larger changes to postural sway kinematics compared to males.

The results showed that all children improved, however contrary to our first prediction, the gymnastics group did not perform better compared to the TPE group or make larger changes to balance performance. These results did not support previous research into the positive effects of motor skill interventions (Bardid et al., 2016; Rudd et al., 2017a,b).

However, in support of the third hypothesis, the GYM group made larger changes to postural stability (i.e., they made larger reductions to sway area) and A/P velocity. Further, in support of the third hypothesis, the changes made to sway area, A/P and M/L sway velocity by GYM group males provides further evidence that gymnastics training for young males has apparent advantages for control of posture such that improvements to postural stability can be made relatively rapidly (i.e., 3 months). Additionally, and in line with previous literature (Garcia et al., 2011), our results suggest (i.e., changes to sway velocity) that even with short periods of gymnastics training, young males may have improved use of sensory information for postural control.

With respect to the last hypothesis, sex appears to have had a particularly influential effect in this study. Sex was revealed as having a main or interaction effect for all dependent variables. For example, independent of the group they were in, only males made significant changes to sway regularity and all participants except TPE males reduced sway area and sway velocity – this was contrary to our fifth hypothesis. These findings demonstrate that when children, and particularly males, are traversing a critical period of perceptual-motor development (Riach and Starkes, 1994; Assaiante and Amblard, 1995; Kirshenbaum et al., 2001; Rival et al., 2005; Austad and van der Meer, 2007) gymnastics may act to mitigate any reduction in postural performance as the recalibration of the postural control system is ongoing. But, despite the changes to postural control demonstrated by the males in the gymnastics group, in support of our fourth prediction and previous research, we found that females were more successful than males at the balancing task (Morris et al., 1982; Raudsepp and Pääsuke, 1995; Venetsanou and Kambas, 2011).

According to one sway regularity model, increased postural control is related to decreased sway regularity (Roerdink et al., 2011). For example, in a static postural control task, compared to novices, experts have relatively irregular COP sway. However, Roerdink et al. (2011) cautioned that the model still had yet to be fully tested with respect to the effects that aging or other organismic, task or environmental constraints may have on sway regularity. While some research has added weight to the idea that with increased postural expertise sway would be expected to be relatively irregular (Lamoth et al., 2009; Stins et al., 2009; Isableu et al., 2017), results from the present study do not support the sway regularity model proposed by Roerdink et al. (2011). In fact, when improvements to performance were seen in the present study, our participants significantly increased sway regularity. Hence, the sway regularity data from the present study suggests that postural sway becomes *more regular* as young children become more proficient at static balance tasks (at least across 3–4 months). A similar trend of increased postural control and increased regularity was also seen in the development of independent sitting in infants (Harbourne and Stergiou, 2003; Deffeyes et al., 2011). It may be the case that infants and young children who are undergoing significant developmental changes act to constrain degrees of freedom in an attempt to simplify the postural task to gain control of their posture. This type of behavioral change would be seen as increased regularity in postural sway. Further, it may be that postural tasks are able to be refined resulting in more irregular sway patterns only after adult-like postural behavior is achieved by older children.

The effect of freeing or freezing degrees of freedom (as increased or decreased regularity indicate, respectively), may be revealed by changes to postural control dynamics (such as sway velocity or sway area). In our study, changes to sway regularity coincided with changes to performance and other kinematic variables. As such our results show that changes to sway regularity may be manifested by changes in sway area or velocity, or vice versa. The present study and the research of Harbourne and Stergiou (2003) and Deffeyes et al. (2011) contribute to an evolving understanding of how postural sway dynamics change as a function of skill particularly among young children.

## Summary and implications

Regardless of the type of physical education children participate in, they are able to increase their ability to balance on one foot across 3 months. Children in both the GYM and TPE groups made changes to control of degrees of freedom and postural control dynamics. Additionally, the GYM group, particularly the males, were able to make changes more rapidly (i.e., across 3 months) compared to the TPE group.

The present study has confirmed that changes in body sway regularity may not necessarily translate directly into postural control, but rather there may be an interaction between changes to sway area and or velocity and sway regularity. However, a limitation of this study was that the children were still in a critical period of perpetual-motor development that is characterized by increased performance variability (Rine et al., 1998; Austad and van der Meer, 2007; Olivier et al., 2007, 2010). Longitudinal tracking of postural development prior to and then after this critical period of development is necessary to confirm either the “U” shaped developmental pathway (*cf.* Newell, 1998) or increasingly regular developmental pathway (*cf.* Schärli et al., 2012) for sway complexity in static postural conditions.

Strengths of the study included the large sample size, the sensitive analysis of COP and the realistic school-based intervention that were deployed. However, a notable finding was high within-group variability and it is possible this feature may have masked differences between groups and sexes. At each phase of the study, the TPE’s group’s 95% confidence intervals for success and kinematic variables were at least 50% wider than those of the GYM group. Additionally, TPE group’s the within-group variability (i.e., relative standard error) for each dependent variable was at least 1.4 times higher than that found in the gymnastics group and these values are similar to previous research (Rine et al., 1998; Olivier et al., 2007). With wide confidence limits and relatively high variability in the TPE group, detecting differences between groups is difficult. Such large within-group variability can mask actual between-phase changes to postural control that may result from training, age, or differences that exist due to sex; where some children made very large improvements to performance or kinematics, others showed large deficits. The expected challenge of analyzing a highly variable cohort was mitigated with the careful design of mixed models prior to performing statistical analysis, as such the effect of high within-group variability in present findings was reduced.

An important limitation of the present study relates to the different teachers providing the intervention and control programmes. Gymnastics lessons were taught by a qualified gymnastics coach who had no prior knowledge of the children they taught. On the contrary, children in the group that completed typical primary school physical education did so under the instruction of their usual teacher. The TPE group teacher’s knowledge of the students may have had an unanticipated positive effect on the educational experience. As such, it may be beneficial for schools to have specialist physical education teachers, that are familiar to students and who are trained to lead gymnastics programmes – as was the case for the TPE group in the present study.

Finally, these results showed that 20 lessons across 10 weeks are sufficient to enable children to make meaningful changes to postural control and balance performance. However, specialist movement education programmes or physical literacy interventions introduced in schools should consider the focus of the activities that children complete. Our findings lead us to propose that Physical Educational curricula should include educational gymnastics because it is characterized by somewhat unique challenging task and postural demands. For example, using the arms to support body weight in dynamic movements and rolling and rotating about various axes, unlike FMS like the typical running, skipping or jumping activities emphasized in sports and traditional physical education curricular content.

## Data availability statement

The raw data supporting the conclusions of this article will be made available by the authors, without undue reservation.

## Ethics statement

The studies involving human participants were reviewed and approved by University of Otago Human Ethics Committee. Written informed consent to participate in this study was provided by the participants’ legal guardian/next of kin.

## Author contributions

NA and CB designed the study. NA conducted data collection. NA, CB, and PL contributed to the data analysis, data presentation, manuscript preparation, and proof-reading. All authors contributed to the article and approved the submitted version.

## Conflict of interest

The authors declare that the research was conducted in the absence of any commercial or financial relationships that could be construed as a potential conflict of interest.

## Publisher’s note

All claims expressed in this article are solely those of the authors and do not necessarily represent those of their affiliated organizations, or those of the publisher, the editors and the reviewers. Any product that may be evaluated in this article, or claim that may be made by its manufacturer, is not guaranteed or endorsed by the publisher.
